# Pattern of antibiotics use, incidence and predictors of surgical site infections in a Tertiary Care Teaching Hospital

**DOI:** 10.1186/s13104-018-3643-8

**Published:** 2018-07-31

**Authors:** Ezaedin Halawi, Tamrat Assefa, Sadikalmahdi Hussen

**Affiliations:** Department of Pharmacology and Clinical Pharmacy, School of Pharmacy, Addis Ababa University College of Health Sciences, P.O.Box:1176, Addis Ababa, Ethiopia

**Keywords:** Pre and postoperative antimicrobial prophylaxis, Tikur Anbessa Specialized Hospital

## Abstract

**Objective:**

Surgical site infections (SSIs) were the most common healthcare-associated infection mainly in developing countries. Inappropriate use of surgical antibiotic prophylaxis, in terms of antibiotic choice, timing, and duration, can lead to the selection of resistant microorganisms and high costs. The aim of this study was to investigate the pattern of antibiotic use, incidence and predictors of SSIs at Tikur Anbessa Specialized Hospital, Addis Ababa, Ethiopia.

**Results:**

From 131 patients, 55.7% were male study participants. Ninety (68.7%) patients received preoperative prophylaxis. Ceftriaxone was the most 76 (84.5%) prescribed agent for prophylaxis. Twenty-seven (20.6%) patients developed surgical site infection. Previous surgery AOR = 3.22 (95% CI [1.14–9.13]) and alcohol use AOR = 7.04 (95% CI [2.56–23.12, p = 0.000]) were independent predictors of SSIs in multivariate logistic regression analysis.

**Electronic supplementary material:**

The online version of this article (10.1186/s13104-018-3643-8) contains supplementary material, which is available to authorized users.

## Introduction

Surgical site infection (SSI) is an infection that happens within 30 days of operation or after 1 year if the implant is placed at or near the surgical incision [1]. It accounts for 17% of all healthcare associated infections and are the second most common hospital-acquired infections in study conducted in Ethiopia [2] and especially during post-operation period [3]. Globally, SSI rates have been found to be from 2.5 to 41.9%. In Africa, SSIs were the leading infections in hospitals (pooled cumulative incidence of 5.6 per 100 surgical procedures), strikingly higher than proportions recorded in developed countries [1] as 13, 20.6, 10.9 and 10.9–75% rate of SSIs were reported in Nigeria [4], Cameron [5], Tanzania [6] and Ethiopia [2, 7] studies respectively.

The extent of microbial contamination at an incision site, host factors (such as age, nutritional status, lifestyle, comorbidities, immune-competency and coexisting infections), the length of the preoperative hospital period, preoperative procedures and the duration and performance of the operation contribute to increased risks of SSIs [8]. Patient characteristics possibly associated with increased risk of SSIs include coincident remote site infections or colonization, diabetes, cigarette smoking, systemic steroid use, obesity (> 20% ideal body weight), extremes of age, poor nutritional status, and perioperative transfusion of certain blood products [9].

SSIs can have a devastating impact on the patient’s course of treatment and is associated with increased treatment intensity, prolonged length of stay and higher costs [10]. A study in the United States of America suggested that programs that reduce the incidence of surgical site infections can substantially decrease morbidity and mortality and reduce the economic burden for patients and hospitals [11]. Despite improvements in operating room practices, instrument sterilization methods, better surgical technique and the best efforts of infection prevention strategies, SSIs remain a major cause of hospital-acquired infections and rates are increasing globally even in hospitals with most modern facilities and standard protocols of preoperative preparation and antibiotic prophylaxis [1].

Another well-documented approach is to use pre and postoperative antimicrobial prophylaxis. From patients that received antimicrobial prophylaxis 30–90% are inappropriate; most antimicrobials are either given at the wrong time, wrong dosage and wrong strength which results in increased antibiotic usage, increased costs, prolonged hospitalization, super infection, antimicrobial resistance and reduction of surgical antimicrobial prophylaxis (SAP) used [12, 13].

Although the high incidence of SSI is suspected in Ethiopia, the magnitude of the problem is not known in TASH. It becomes therefore essential to determine the pattern of antibiotic use in surgical wards and occurrence and predictors of SSIs.

## Main text

### Methods

#### Study setting

The study was conducted in Tikur Anbessa Specialized Hospital (TASH) Addis Ababa, Ethiopia. TASH is one of the largest tertiary level, referral and teaching hospitals in the country, affiliated to Addis Ababa University. TASH has approximately 570 beds and out of this, 150 are allocated to surgical patients.

#### Study design and period

A prospective observational study was undertaken from April 1 to April 30, 2017. The source population was all inpatients admitted to the hospitals and undergone surgery, whereas the study population was all patients who had undergone operation and admitted to surgical ward during the study period.

#### Inclusion and exclusion criteria

All surgical patients who had operated during the study period and hospitalized up to 30 days were considered as eligible for the study. However, patients from other wards of the hospital (internal medicine, intensive care unit, emergency) and patients who died or left before third postoperative days and those not voluntary to participate were excluded from the study.

#### Sample size and sampling technique

Convenient sampling was used in as sampling technique in this study. Accordingly, all patients who had undergone surgical procedures are followed from admission until discharge, including any readmissions for infection. Accordingly, 131 patients were enrolled in our study.

#### Data collection, management and quality assurance

Socio-demographic characteristics of the patients, surgery-related information(site of surgery, duration of surgery, previous history of surgery, surgery type, hospital stay after surgery, wound class and occurrence of SSI after surgery within 30 days), antibiotic used (preoperative and postoperative antibiotic used, duration of antibiotics after surgery) were collected using data abstraction tool from patient’s medical chart. The tool was developed from literatures and modified at it suits to study setting up and senior clinical pharmacist was consulted for its appropriateness. Patients were interviewed in case the information couldn’t be obtained from their chart to make the data complete. Antimicrobial use evaluation was done according to Center for Disease Prevention and Control (CDC) criteria for using antimicrobials in surgical site infection (SSI) prevention and treatment [9]. In our set up, SSI diagnosed mainly by interpretation of clinical (sign and symptom manifestation) findings and also incorporating CDC definition and methodology. It is rare to use laboratory results (culture testing) in diagnosing SSI in the studied hospital. Incidence and risk factors for SSI, including those for specific procedures were calculated from data collected on daily base. Before the actual data was collected, a pretest of the data collection instrument was done to ensure the appropriateness of questionnaire and necessary modifications were made.

#### Data entry and analysis

Data was entered into and analyzed by SPSS version 20. Logistic regression analysis was used to identify predictors of SSI. In all cases, p-value less than 0.05 are taken as statistically significant. Variables which have a significant association at p-value < 0.25 in the bivariate logistic regression analysis were candidates for multivariate logistic regression analysis to identify predictors of SSIs.

#### Ethical considerations

Ethical clearance was obtained from the ethics review committee of School of Pharmacy, College of Health Sciences, Addis Ababa University. Additionally written consent was taken from each participant before participating in the study.

### Results

#### Socio-demographic characteristics

During the study period, a total of 158 patients underwent surgery in selected wards of the hospital. From the total, 27 patients were excluded by exclusion criteria (refused to provide consent or death or discharged too early after surgery) and finally, 131 patients were considered for analysis. The age of the patients ranged from 2 to 86 years with a mean of 41.15 ± 18.29 years. Twenty-two (16.8%) and 41 (31.3%) patients were smokers and alcohol drinkers respectively (Table 1).

**Table 1:** Socio-demographic and clinical characteristics of patients at surgical wards of TASH (N = 131)

Socio-demographic and clinical characteristics	N (%)
Gender	
Female	58 (44.3)
Male	73 (55.7)
Age in years	
< 30	39 (29.8)
30–50	40 (30.5)
> 50	52 (39.7)
Residence	
Urban	71 (54.2)
Rural	60 (45.8)
Cigarette smoking	
No	109 (83.2)
Yes	22 (16.8)
Alcohol intake	
No	90 (68.7)
Yes	41 (31.3)
Preoperative blood transfusion	
No	101 (77.1)
Yes	30 (22.9)
Systemic steroid use	
No	115 (87.8)
Yes	16 (12.2)
Preoperative hospital stay (days)	
> 7	62 (47.3)
≤ 7	69 (52.7)
Co-morbidities	
Cardiovascular disease	20 (15.3)
Hypertension	9 (6.9)
Diabetes mellitus	2 (1.5)
HIV/AIDS	5 (3.8)
Infection^a^	8 (6.1)
Multiple co-morbidities	7 (5.3)
Others^b^	6 (4.6)
Wound class	
Clean	43 (32.8)
Clean contaminated	64 (48.9)
Contaminated	16 (12.2)
Dirty	8 (6.1)
Surgery type	
Emergency	25 (19.1)
Elective	106 (80.9)
Previous surgery	
No	98 (74.8)
Yes	33 (25.2)

Infection^a^: infections other than SSIs. Others^b^ include cancer, psychotic disorders, asthma, and epilepsy

#### Clinical characteristics and co-morbidities of the patients

Nearly half of patients (47.3%) stayed in hospital for more than 7 days before surgery was conducted. Fifty-seven (43.5%) of the study participants had various types of co-morbidities beside their surgery indications. Six (4.6%) of them had more than co-morbidities (Table 1). About half of patients (48.9%) who underwent operation had clean contaminated wounds at the time of surgery and the mean duration of operation was 2.21 ± 1.53 h. One hundred six (80.9%) of the operations were elective surgery. One-fourth of patients had a history of previous procedure (Table 1).

#### Practice and appropriateness of surgical antimicrobial prophylaxis

Among 131 patients, more than two-third of (68.7%) patients received preoperative antimicrobial prophylaxis. Out of the 43 and 64 patients who had a clean and clean contaminated wound, 15 (34.9%) and 58 (90.6%) patients received pre-operative antimicrobial prophylaxis respectively. From all patients that took preoperative antimicrobial prophylaxis eighty (88.9%) patients received antimicrobial prophylaxis for greater than 24 h after surgery. The majority of patients received ceftriaxone 76 (84.5%). The most commonly prescribed regimen among the combination regimens was ceftriaxone plus metronidazole 12 (13.3%) (Table 2).

**Table 2:** Practice and appropriateness of surgical antimicrobial prophylaxis in surgery patients in TASH (N = 131)

Practice of antimicrobial prophylaxis(AMP)	N (%)
Preoperative AMP provision	
Yes	90 (68.7)
No	41 (31.3)
Preoperative provided antibiotics	
Ceftriaxone	76 (84.5)
Ceftriaxone and metronidazole	12 (13.3)
Ampicillin	2 (2.2)
Time of administration of preoperative AMP (h)	
≤ 1	34 (37.8)
>1	56 (62.2)
Duration of postoperative AMP (h)	
≤ 24	10 (11.1)
> 24	80 (88.9)
Indication of AMP	
Indicated and administered	73 (91.25%)
Not indicated and not administered	34 (66.7%)
Indicated but not administered	7 (8.75%)
Not indicated but administered	17 (33.3%)

#### Incidence of surgical site infections

Among a total of 131 patients who underwent surgical procedures, SSI was developed in 27 (20.6%) patients before discharge. Among the detected SSIs, 23 (85.2%) and 4 (14.8%) were superficial and deep infections respectively.

#### Factors associated with surgical site infections

Bivariate logistic regression model showed seven variables were associated with the occurrence of SSIs at p < 0.25. Alcohol (p = 0.000), cigarette smoking (p = 0.05), preoperative blood transfusion (p = 0.05), contaminated (p = 0.17) and clean wound (p = 0.12), previous surgery (p = 0.01), and duration of surgery (p = 0.20) were candidate variables for multivariate analysis. The remaining factors were not associated with SSIs in studied participants (see Additional file 1).

#### Predictors of surgical site infections

Upon further analysis using multivariate analysis to evaluate whether these variables are independent predictors of SSIs, alcohol use and previous surgery were found to be independently associated with development of SSIs (AOR = 7.70, p = 0.000), (AOR = 3.22, p = 0.028). However, other socio-demographic and clinical characteristics were not independently predictors of SSIs (Table 3).

**Table 3:** Predictors of SSI development in surgery patients in TASH (N = 131)

Variables	Surgical site infection		COR (95% CI)	AOR (95% CI)	p value
	Yes N (%) 27 (20.6)	104 (79.4%)			
Preoperative blood transfusion					
Yes	10 (33.3%)	20 (66.7%)	2.47 (0.98–6.21)	1.34 (0.45–4.02)	0.602
No	17 (16.8%)	84 (83.2%)		1	
Cigarette smoking					
Yes	8 (36.4%)	14 (63.6%)	2.71 (0.1–7.36)	0.80 (0.23–2.80)	0.725
No	19 (17.4%)	90 (82.6%)		1	
Alcohol					
Yes	18 (43.9%)	23 (56.1%)	7.04 (2.78–17.75)	7.70 (2.56–23.13)	0.000
No	14 (14.74%	81 (85.26%)		1	
Wound class					
Clean	6 (13.95%)	37 (86.05%)	0.27 (0.05–1.44)	0.53 (0.07–4.08)	0.543
Clean contaminated	16 (25%)	48 (75%)	0.56 (0.12–2.59)	1.04 (0.16–6.57)	0.966
Contaminated	2 (12.5%)	14 (97.5%)	0.24 (0.03–1.87)	0.67 (0.06–7.24)	0.745
Dirty	3 (37.5%)	5 (62.5%)		1	
Previous surgery					
Yes	12 (36.4%)	21 (63.6%)	3.16 (1.29–7.76)	3.22 (1.14–9.13)	0.028
No	15 (15.3%)	83 (84.7%)		1	
Duration of surgery (h)					
> 1	19 (24.1%)	60 (75.9%)	1.81 (0.73–4.51)	2.16 (0.72–6.48)	0.169
≤ 1	8 (15.4%)	44 (84.6%)		1	

*AOR* adjusted odds ratio, *COR* crude odds ratio

### Discussion

In this study, majority of patients who underwent surgical procedures received antimicrobial prophylaxis which is in line with another study [12]. However, proper AMP practice in this setup was not parallel with recommendations of the clinical practice guideline for antimicrobial prophylaxis in surgery [14, 15].

In this study, none of the patients received cefazolin even if it is recommended by guideline [14] in the prevention of most surgery related infections. The most commonly prescribed drug for AMP was ceftriaxone, followed by metronidazole, and it is comparable to study done in Brazil [16]. For surgical prophylaxis, it is important to select an antimicrobial with narrowest antibacterial spectrum to reduce the emergence of resistance and as for covering the most likely contaminating microorganisms for that type of surgery [14]. In all patients that received antimicrobial prophylaxis selection of antimicrobials were not consistent with the recommendations of the guidelines which may be due to the absence of their own guideline, the assumption of ceftriaxone similar with cefazolin or unavailability of first generation cephalosporins.

Administration of AMP should be within 1 h prior to incision to achieve adequate protection [17]. In our study, only 37.8% of patients received preoperative antibiotics within 1 h prior to incision which is in agreement with Brazilian study [16]. However, AMP administration time is not recorded in 17 (18.9%) patients may be due to work overload on attending nurses, the absence of separate sheet for recording time of administration and lack of awareness to record AMP administration time which is consistent with other studies [12].

Results from other study showed that duration of AMP was longer than 24 h in 30–90% of cases after surgery [18] and similarly this was observed in nearly 90% of our study participants. In general, single-dose prophylaxis or prophylaxis ending within 24 h after operation is recommended by guidelines [14]. Prolonged postoperative dosing of antibiotics does not provide additional benefits and is associated with increased risk of adverse events and induction of antimicrobial resistance [14, 15].

In most part of the world depending on the set up of their hospitals and their degree of adherence to aseptic techniques, the SSI rate has varied from a low of 2.5% to a high of 41.9% [19–22]. The incidence rate of SSIs found in the present study was 20.6%. It was higher than previous findings from developing country hospital 14.8% [23] and from USA 7.2% [24]. However, SSIs rate was similar to the study done in India 20.09% [25], and Nigeria 20.3% [26] and Ethiopia 19.1% [1]. But, it was more than three times lower when compared with the study conducted in Ethiopia 75% [7]. The elevated SSI rates can be explained by the using clinical evidence than laboratory in the studied set up to detect SSI and limited ventilation in the operating theatre, as well as limited application of infection control measures.

In this study, alcohol use and having previous surgery was significant predictors of postoperative SSIs. The present study showed that patients with drinking alcohol were 7.70× more likely to develop SSIs compared with patients who do not drink alcohol with AOR = 7.04 (95% CI [2.56–23.12, p = 0.000]), and another study reported similar association [3]. Patients who had history of previous surgery were 3.16 times more likely to develop SSIs compared with those who hadn’t previous surgery with AOR = 3.22 (95% CI [1.14–9.13]), which was in agreement with another study [27]. Higher incidence of SSI in patients with previous surgery may be due to exposure to long operation time, difficult surgery, high class wounds and using the same incision site with the previous one.

Some studies have examined the use of the ASA score as a predictor of SSI risk [20, 28]. The ASA score approximates ‘global’ patient health at the time of the operation and is a reliable predictor of the risk of SSI [29]. This may be because these procedures were mainly performed on relatively normal physical activity before admission, which may limit the utility of the ASA score as a discriminator of risk. According to this study emergent surgeries (24%) had higher rates of SSI than elective procedures (19.8%). This is because, as emergency operations should be higher risk because of suboptimal preoperative preparation and because they are more likely to be dirty.

## Limitations

We used smaller sample size and shorter study period which might have an impact on some of the results reported. Other factors have been known to influence the risk of SSIs, such as operation characteristics and nutritional status, antiseptic usage, and sterilization techniques and quality of operation theatre was not studied in this study.

## Additional file

**Additional file 1.** Factors associated with SSIs occurrence among surgical patients in TASH (N = 131). It is additional material which describes bivariate logistic regression model showing seven variables were associated with the occurrence of SSIs at p < 0.25. Alcohol (p = 0.000), cigarette smoking (p = 0.05), preoperative blood transfusion (p = 0.05), contaminated (p = 0.17) and clean wound (p = 0.12), previous surgery (p = 0.01), and duration of surgery (p = 0.20) were candidate variables for multivariate analysis. The remaining factors were not associated with SSIs development in studied participants.

## Abbreviations

AMP: antimicrobial prophylaxis
AOR: adjusted odds ratio
SAP: antimicrobial prophylaxis
SSI: surgical site infection
TASH: Tikur Anbessa Specialized Hospital
