# Supplementary material for: Pattern of antibiotics use, incidence and predictors of surgical site infections in a Tertiary Care Teaching Hospital
Source: BMC Res Notes. 2018 Jul 31;11:538. doi: 10.1186/s13104-018-3643-8 (PMC6069967; doi:10.1186/s13104-018-3643-8)
Supplement: Supplementary file 1 — Additional file 1. Factors associated with SSIs occurrence among surgical patients in TASH (N = 131). It is additional material which describes bivariate logistic regression model showing seven variables were associated with the occurrence of SSIs at p < 0.25. Alcohol (p = 0.000), cigarette smoking (p = 0.05), preoperative blood transfusion (p = 0.05), contaminated (p = 0.17) and clean wound (p = 0.12), previous surgery (p = 0.01), and duration of surgery (p = 0.20) were candidate variables for multivariate analysis. The remaining factors were not associated with SSIs development in studied participants. [file 13104_2018_3643_MOESM1_ESM.docx]

Factors associated with SSIs occurrence among surgical patients in TASH (N=131)

| **Variables** | **Surgical site infection** | |  |  |  |
| --- | --- | --- | --- | --- | --- |
|  | **Yes N (%)**  **27(20.6)** | **No N (%)**  **104(79.4%)** | **Crude OR** | **95%CI** | **P value** |
| Gender |  |  |  |  |  |
| Male | 15(20.5%) | 58(79.5%) |  | 1 |  |
| Female | 12(20.7%) | 46(79.3%) | 1.01 | 0.43-2.36 | 0.98 |
| Residence |  |  |  |  |  |
| Urban | 16(22.5%) | 55(77.5%) | 1.30 | 0.55-3.06 | 0.56 |
| Rural | 11(18.3%) | 49(81.7%) |  | 1 |  |
| Age in years |  |  |  |  |  |
| <30 | 5(12.8%) | 34(87.2%) | 0.59 | 0.17-1.99 | 0.39 |
| 30-50 | 8(20%) | 32(80%) |  | 1 |  |
| >50 | 14(26.9%) | 38(73.1%) | 1.47 | 0.55-3.96 | 0.44 |
| Preoperative blood transfusion |  |  |  |  |  |
| Yes | 10(33.3%) | 20(66.7%) | 2.47 | 0.98-6.20 | 0.05 |
| No | 17(16.8%) | 84(83.2%) |  | 1 |  |
| Systemic steroid use |  |  |  |  |  |
| Yes | 4(25%) | 12(75%) | 1.33 | 0.39-4.52 | 0.644 |
| No | 23(20%) | 92(80%) |  |  |  |
| Cigarette smoking |  |  |  |  |  |
| Yes | 8(36.4%) | 14(63.6%) | 2.71 | 0.1-7.36 | 0.051 |
| No | 19(17.4%) | 90(82.6%) |  | 1 |  |
| Alcohol |  |  |  |  |  |
| Yes | 18(43.9%) | 23(56.1%) | 7.04 | 2.79-17.75 | 0.000 |
| No | 9(10%) | 81(90%) |  | 1 |  |
| ASA score |  |  |  |  |  |
| <=1 | 11(18%) | 50(82%) |  | 1 |  |
| >1 | 16(22.9%) | 54(77.1%) | 1.35 | 0.57-3.18 | 0.497 |
| Preoperative hospital stay |  |  |  |  |  |
| >7 days | 14(22.6%) | 48(77.4%) | 1.26 | 0.54-2.93 | 0.598 |
| <=7 days | 13(18.8%) | 56(81.2%) |  | 1 |  |
| Co-morbidities |  |  |  |  |  |
| Yes | 10(20.8%) | 38(78.2%) | 1.02 | 0.42-2.48 | 0.962 |
| No | 17(20.5%) | 66(79.5%) |  | 1 |  |
| Wound class |  |  |  |  |  |
| Clean | 6((13.95%) | 37(86.05%) | 0.27 | 0.05-1.44 | 0.125 |
| Clean contaminated | 16(25%) | 48(75%) | 0.56 | 0.12-2.59 | 0.454 |
| Contaminated | 2(12.5%) | 14(97.5%) | 0.24 | 0.03-1.87 | 0.172 |
| Dirty | 3(37.5%) | 5(62.5%) |  | 1 |  |
| Surgery type |  |  |  |  |  |
| Emergency | 6(24%) | 19(76%) | 0.78 | 0.28-2.2 | 0.642 |
| Elective | 21(19.8%) | 85(80.2%) |  | 1 |  |
| Previous surgery |  |  |  |  |  |
| Yes | 12(36.4%) | 21(63.6%) | 3.16 | 1.29-7.76 | 0.012 |
| No | 15(15.3%) | 83(84.7%) |  | 1 |  |
| Duration of surgery |  |  |  |  |  |
| >1hour | 19(24.1%) | 60(75.9%) | 1.81 | 0.73-4.51 | 0.202 |
| <=1hour | 8(15.4%) | 44(84.6%) |  | 1 |  |
| Antimicrobial prophylaxis |  |  |  |  |  |
| Yes | 18(20%) | 72(80%) | 0.89 | 0.36-2.11 | 0.798 |
| No | 9(21.2%) | 32(78.1%) |  |  |  |
